# The Molecular Pages of the mesotelencephalic dopamine consortium (DopaNet)

**DOI:** 10.1186/1471-2105-5-174

**Published:** 2004-11-01

**Authors:** Nicolas Le Novère, Marco Donizelli

**Affiliations:** 1 Computational Neurobiology, EMBL-EBI, Wellcome-Trust Genome Campus, Hinxton Cambridge, CB10 1SD UK

## Abstract

**Background:**

DopaNet  is a Systems Biology initiative that aims to investigate precisely and quantitatively all the aspects of neurotransmission in a specific neuronal system, the mesotelencephalic dopamine system. The project should lead to large-scale models of molecular and cellular processes involved in neuronal signaling. A prerequisite is the proper storage of knowledge coming from the literature.

**Methods:**

DopaNet Molecular Pages are highly structured descriptions of quantitative parameters related to a specific molecular complex involved in neuronal signal processing. A Molecular Page is built by maintainers who are experts in the field, and responsible for the quality of the page content. Each piece of data is identified by a specific ontology code, annotated (method of acquisition, species, etc.) and linked to the relevant bibliography. The Molecular Pages are stored as XML files, and processed through the DopaNet Web Service, which provides functionalities to edit the Molecular Pages, to cross-link the Pages and generate the public display, and to search them.

**Conclusions:**

DopaNet Molecular Pages are one of the core resources of the DopaNet project but should be of widespread utility in the field of Systems Neurobiology.

## Background

Although the use of Systems Biology to understand the function of neurons recently started to gain momentum, it is still in its infancy. A crucial step leading to meaningful simulations is the reconstruction of large neuronal systems based on the elementary building blocks. However, this approach suffers from the lack of truly quantitative values. Some projects of functional genomics have been recently launched to try to remedy the problem, e.g. the *Genes to Cognition *consortium ([1,2]). Another problem impairing the integration process resides in the large diversity of protocols and model systems used to gather the data. Since a large proportion of the data is potentially shared by numerous different systems, it is sensible to focus the effort on a restricted and well delineated system. This approach has been pioneered by the *Alliance for Cellular Signaling*, launched by Alfred Gilman ([3,4]), which originally focused on the B-lymphocyte and the cardiac myocyte.

### DopaNet

Similarly, we started at the end of 2001 the mesotelencephalic dopamine consortium (DopaNet, [5]). The initiative aims to investigate precisely and quantitatively all the aspects of neurotransmission – at the levels of the molecule, the supra-molecular assembly, the neuronal cell and the neuronal network – in a specific neuronal system involved in many neuropathologies, such as Parkinson's disease, schizophrenia and drug addiction [6-8]. The resulting integrated knowledge will not only provide relevant, up-to-date information about such pathologies, but will also form a firm substrate to link the function of the neurobiological structures and the implementation of cognitive and mental abilities. As of June 2004, 35 European teams from 8 countries are part of the project. DopaNet became a network of the European Science Foundation (ESF) in January 2003.

A first step, prior to the design of large-scale dedicated experiments, consists in data mining the current literature in molecular and cellular neurobiology for existing quantitative knowledge. The resulting data has to be properly stored and annotated.

### DopaNet Molecular Pages

A DopaNet Molecular Page is a collection of annotated numerical data relative to a "molecular complex" present in one or several DopaNet target cells. The "molecular complex" is taken here in the sense of the DopaNet Neuronal Ontology (see below), as a "stable assembly of molecules", a "molecule" being described as a "set of atoms linked together by covalent bounds". As a consequence, we can have a Molecular Page storing data relative to a molecular complex made up of components, that are themselves described in other Molecular Pages. An anticipated example is an heterotrimeric G protein and its *α *and *βγ *subunits. The information collected deals with the structure of the complex, its anatomical distribution within DopaNet target cells, and its functional properties. Each page is under the responsibility of its maintainer(s), who decide which data is to be included or not, and acknowledge the input of the various contributors. All the data included in a Molecular Page is annotated (Species, Methods, variability etc.), and linked to bibliographic references. In addition, each single data stored in the databases of DopaNet is attached to one or several terms of the DopaNet Neuronal Ontology. This ontology will therefore act as a glue, relating the various pieces of data one to the other.

### DopaNet Neuronal Ontology

An ontology is defined here in its information science meaning, as a hierarchical structuring of knowledge. In our case, it is a relational vocabulary, that is a set of terms linked together, aiming to describe a neuron. Each term has a definition and a unique identifier. Terms are related by "is a" inheritances, which represent sub-classing, and "part of" inheritances which represent deepening knowledge. For instance, the nicotinic receptor subunit alpha6 "is a" nicotinic receptor subunit, and is "part of" the (alpha6)_2_(beta2)_3 _nAChR. Each term can be the child of several others. Therefore the complete picture is not a genealogical tree, but rather a network or relationships.

There are several biological ontologies, the most famous (and complete) being Gene Ontology ([9,10]). Numerous other projects can be found at repository of the Open Biological Ontologies ([11]). See the discussion below for the relation between Gene Ontology and DopaNet Neuronal Ontology. In DopaNet Neuronal Ontology, a "molecular complex" is defined as a "stable assembly of molecules". An obvious example of a molecular complex is a protein. A molecular complex contains one or several components. Those components are all "molecule", a molecule being defined as a "set of atoms linked together by covalent bounds". For instance, "(alpha4)_2(beta2)_3 nAChR" is a "nicotinic acetylcholine-gated receptor". It is made up of two components: "alpha4 nicotinic receptor subunit" and "beta2 nicotinic receptor subunit", that are present in the "molecule" subtree as "nicotinic receptor subunit". The "polypeptide" subtree of "molecule" is built following the sequence resemblances, and then the 3D structure, similarly to the Structural Classification Of Protein database ([12,13]) and InterPro ([14,15]). Contrary to the "molecular complex" branch, the "molecule" branch should not contain any group based on the function.

## Construction and content

### Molecular page structure

A Molecular Page is made up of a header followed by several lists, each list containing a sequence of identical elements. There are currently twelve main lists, described below. Several other lists of items are used to described the page data at a finer level. According to the molecular complex described in the page, some of the lists can be empty.

The Molecular Page header contains the name of the molecular complex described in the page, an abbreviation, the unique ontology code used to identify the page, the dates of creation and last modification of the page, and the page status. The possible status are:

#### stable

The Molecular Page has been submitted by the maintainers and is ready for public release.

#### unstable

A new version of the Molecular Page, not yet ready for public release.

#### forthcoming

A new Molecular Page in construction, that has never been submitted for public release.

For instance, the nicotinic acetylcholine receptor (*α*4)_2_(*β*2)_3 _is described in the Page describing the complex "(alpha4)_2(beta2)_3 nAChR" . The related Ontology term can be found at 

<molecularPage DopaNetontology="DA:0000027"

                         abbreviation="nAChRa4_2b2_3"

                         creation="2003-01-05T00:00:00"

                         modification="2004-09-02T15:49:41"

                         name="(alpha4)_2(beta2)_3 nAChR"

                         status="stable">

The main lists that compose a Molecular Page are:

#### List of maintainers

Maintainers are the only people authorized to directly modify the Molecular Pages. They are responsible for the quality and the completeness of the data included in the Page. However, maintainers are not assumed to systematically gather the information all by themselves. They are encouraged to contact experts to help them. Helpful people should be acknowledged as contributors.

#### List of contributors

Contributors are all the people who bring new information about a Molecular Page, or correct an existing piece of information. Contributors can be seen as the equivalent of authors of an article. Except maintainers (who are contributors by definition), they cannot directly modify a Molecular Page. They have to contact a maintainer instead. Note that the database administration team can directly modify the Molecular Pages to comply with the guidelines.

#### List of components

A Molecular Page describes a molecular complex. This complex is made up of components (at least one). The listOfComponents describes those components, their stoichiometry, and lists useful related resources. Each component is annotated by its ontology code.

The complex "(alpha4)_2(beta2)_3 nAChR" [DA:0000027] is made up of two components, the subunit *α*4 and the subunit *β*2.

 <listOfComponents>

  <component DopaNetontology="DA:0000188"

                     name="alpha4 nicotinic receptor subunit"

                     stoichiometry="2">

   <listOfResources>

     <resource identifier="ACHa4hosa"

                    name="Ligand-Gated Ion Channel database"

                    references="1"

                    url="">

           <taxon>Homo sapiens</taxon>

        </resource>

     </listOfResources>

  </component>

 </listOfComponents>

#### List of states

The function of a molecular complex is most often modulated by permutations between various states (conformational transitions, covalent modifications etc.). Accordingly, most of the quantitative data are actually relevant only for one state or a subset of states. Those states should therefore be listed, described and annotated. The quantitative data described in the "functional" lists (see below) will refer both to the states of the molecular complex itself, listed here, and the list of states of other relevant Molecular Pages.

The complex "(alpha4)_2(beta2)_3 nAChR" may exist under (at least) three different states: "basal", "active", and "desensitized".

  <listOfStates>

        <state identifier="basal" name="basal">

                 <description>

                 In the basal state, the ionic pore is closed. This state displays a weak affinity for agonists such as acetylcholine or nicotine.

                 </description>

       </state>

  </listOfStates>

#### List of generic properties

A list of properties that depends solely on the molecular complex itself, and not on its relationships with other entities, such as ligands or substrates. Example of such properties are molecular weight or Stoke radius.

  <listOfGenericProperties>

        <property name="MW" stateMolecule="basal">

            <taxon>Homo sapiens</taxon>

                <listOfValues>

                        <value mean="310971" unit="Dalton">

                             <comment>without covalent modifications.</comment>

                        </value>

                 </listOfValues>

        </property>

  </listOfGenericProperties>

#### List of cells

The distribution of the molecular complex and its components is described within the relevant DopaNet target cells: cortical glutamatergic pyramidal neuron, mesencephalic dopaminergic neuron, striatal cholinergic interneuron, striatal enkephalinergic/GABAergic medium spiny neuron, striatal substance p/GABAergic medium spiny neuron. It is likely that a *listOfExtracellular *shall be necessary at some point.

Each cell is divided into compartments, where the distribution of transcripts and molecules can be described. The approach used to explore the distribution is specified, since both the accuracy and the quantitativeness of the observations strongly depends on the method chosen. As for all the following data, the species where the study has been conducted is also mandatory.

One entry in the complex "(alpha4)_2(beta2)_3 nAChR" is the fact that in the cell soma of the rat mesencephalic dopaminergic neuron, single cell RT-PCR experiments showed that *α*4 is present in 100% of neurons and *β*2 is probably also present in 100% of neurons.

  <listOfCells>

    <cell cellName="mesencephalic dopaminergic neuron" DopaNetontology=" DA:0000702">

        <listOfCompartments>

            <compartment DopaNetontology="DA:0000137" name="cell soma">

               <listOfTranscripts>

                    <transcript method="single cell RT-PCR" references="17">

                          <taxon>Rattus norvegicus</taxon>

                           <description>

                                a4 is present in 100% of neurons. b2 is probably also present in 100% of neurons.

                           </description>

                    </transcript>

              </listOfTranscripts>

           </compartment>

       </listOfCompartments>

  </cell>

</listOfCells>

#### List of ligands

The ligands of a molecular complex are molecules or ions that bind to it. The size of the ligand relative to the molecular complex is irrelevant. Within the Molecular Page of "transforming growth factor receptor type I", one ligand is "transforming growth factor betal". Conversely, in the Molecular Page of "transforming growth factor beta1", one ligand is "transforming growth factor receptor type I"! See table 1 for an example of receptor-ligand reversion. The endogenous ligands are identified by their ontology code.

Functional parameters such as *k*_*on*_, *k*_*off *_or *K*_*m *_can be stored in a controlled manner, in order to be easily retrieved later. Whenever possible, the quantitative values are related to the states of the molecular complexes involved, not only the state of the molecular complex subject of the Molecular Page but also the state of the ligand. This remark holds for the substrates, the translocators and the modulated substances as well (see below). See table 1 for an illustration of the use of state references.

The desensitized "(alpha4)_2(beta2)_3 nAChR" of the rat binds acetylcholine with a Ki versus the epibatidine of 8.6 ± 1.98 nM.

  <listOfLigands>

      <ligand DopaNetontology="DA:0000184"

                     name="acetylcholine"

                     origin="endogenous">

          <listOfProperties>

             <property name="Ki_epibatidine"

                            references="10 18"

                            stateMolecule="desensitized">

                 <taxon>Rattus norvegicus</taxon>

                 <listOfValues>

                     <value mean="8.6" sd="1.98" unit="nanomole per litre"/>

                 </listOfValues>

             </property>

          </listOfProperties>

      </ligand>

  </listOfLigands>

#### List of substrates

All substances modified as a result of an interaction with the molecular complex. The parameters stored here are for instance *K*_*m*_, *k*_*cat *_or *V*_*max*_.

#### List of translocators

The translocators are substances that go from one subcellular compartment to another, the translocation being mediated by the molecular complex. Typical parameters are conductance or relative permeability.

The active complex "(alpha4)_2(beta2)_3 nAChR" of the rat translocates cations with a conductance of 13.3 ± 1.5 pS.

  <listOfTranslocators>

        <translocator DopaNetontology="DA:0000264"

                             name="cation"

                             origin="endogenous">

          <listOfProperties>

             <property name="conductance" references="14" stateMolecule="active">

                  <taxon>Rattus norvegicus</taxon>

                  <listOfValues>

                       <value mean="13.3" sd="1.5" unit="picosiemens"/>

                  </listOfValues>

             </property>

         </listOfProperties>

        </translocator>

  </listOfTranslocators>

#### List of modulated

In many case, one knows about the effect of a molecular complex on a substance, without knowing the detailed mechanism of action. The *modulated *entries are to be avoided as much as possible, since they generally reflect a set of binding and/or enzymatic events.

#### List of transitions

Possible conversions between the states described in the listOfStates, such as a conformational transition, or a covalent modification.

The complex "(alpha4)_2(beta2)_3 nAChR" undergoes conformational transitions between the basal and active states.

  <listOfTransitions>

        <transition state 1="basal" state2="active">

            <comment>

                              In the absence of ligand, the equilibrium is strongly displaced toward the basal state. Agonists, such as acetylcholine and nicotine, stabilise the active state and shift the equilibrium. The transition from basal to active corresponds to an opening of the ionic pore.

            </comment>

        </transition>

  </listOfTransitions>

#### List of bibitems

The list of bibliographic resources used to gather and annotate the data. Each piece of data included in the Molecular Page should be linked to those bibliographic items by internal references.

### Molecule Page storage

Molecular Pages are saved as XML files [16], and their structure is described by an XML schema [17] available at 

Molecular Pages XML files are stored within two different repositories, depending on the status of the Page. One repository contains only the stable Pages ready for public release (22 pages as of October 21, 2004), while the other repository contains also the unstable and the forthcoming Pages (39 pages as of October 21, 2004).

In addition to the two XML repositories, there is also a third HTML repository, containing the human-readable HTML versions of the stable Pages, automatically generated from their XML counterparts using XSL Transformations [18] with the Xalan processor [19]. Based on the identifier attributes, this processing generates the links within the Molecular Pages, but also between different, although related, Molecular Pages.

### Processing

As described above, Molecular Pages are continuously modified and updated by the maintainers, with the help of the contributors. In order to automatize, safe-guard and simplify as much as possible the work required by a maintainer to create and edit a Molecular Page, an application called the *DopaNet Web Service *has been designed and implemented, which provide functionalities to:

1. authenticate Page maintainers

2. browse pages by maintainers

3. grant exclusive Page editing rights to a maintainer

4. create and edit a Page via a rich user interface

5. save or submit the edited Page, setting the "stable/unstable" status

The DopaNet Web Service is made of both server-side and client-side components, all written in Java, and communicating via either the SOAP [20] or the HTTP protocol. The server is deployed into an Apache Tomcat server [21], while the clients are both a Java Applet and a collection of dynamic (Java Server Pages) and static (HTML) pages. The Applet provides a very rich interface to edit a Molecular Page, but due to the Applet technology limitations (security sandbox, download time, etc.), a form-based HTML Page editor is current under development. Most of the Applet and the HTML editor components are derived directly from the DopaNet Molecular Page XML schema, using a mapping between XML schema types and Java GUI or HTML form widgets. Both server- and client-side, Molecular Page XML data is handled using Apache tools such as the Xerces parser [22] and the Xalan processor.

In addition to support the remote creation and editing of Molecular Pages, the DopaNet Web Service provide also functionalities to:

1. register new DopaNet contributors

2. update existing DopaNet contributor information

3. browse Molecular Pages by status

4. search Molecular Pages

Searching of Molecular Pages is implemented by using the API provided by the Apache Xindice [23] native XML database, which has proved to be adequate in terms of speed for the amount of data we currently have in DopaNet.

## Utility and discussion

Although in their early stage of development, DopaNet Molecular Pages provide a unique source of structured, annotated quantitative data about the molecules involved in neuronal signaling. They will feed both the experimental biologist and the theoretician with the best available estimates for all kind of knowledge, whether biochemical, anatomical or functional. This will allow them to design better experiments or formal models, and to benchmark their results. As a side-effect triggered by the mandatory annotations, DopaNet Molecular Pages will also a bibliographic resource, each page being the equivalent of a small review of the literature.

### DopaNet Neuronal Ontology

Gene Ontology is now a fully grown project, and is being widely used in several biological domains. Nevertheless, in its present form, Gene Ontology was not found suitable to be directly used by the DopaNet project. We hope to collaborate with Gene Ontology maintainers in the future. In particular, effort will be made to complete Gene Ontology in the area of Neurobiology. However, DopaNet Neuronal Ontology will never actually be a subset of Gene Ontology. Indeed, the purpose of the latter is to classify the gene products – and one of its most useful application so far has been the annotation of sequence database entries. The purpose of DopaNet Ontology is broader in term of knowledge, and not limited to the classification of gene products. At the same time it is focused onto a specific system, and therefore of interest for a narrower audience.

The Gene Ontology consortium defined three different vocabularies *molecular function*, *biological process*, and *cellular component*. Only the latter is at the moment relevant to DopaNet purposes, that is the Molecular Pages. However, it is anticipated that the *biological process *vocabulary will be needed in the near future, for instance to annotate electrophysiological data. DopaNet *cellular component *vocabulary is larger than Gene Ontology one, since it contains the different kinds of neuronal cells (see the Cell Type ontology [24]) In addition, one can foresee the need of other types of vocabularies to handle more integrated information such as mutant phenotypes, for instance *Molecular function *or vocabularies dealing with behaviors (other efforts have already started in that direction, see for instance the *Mammalian Phenotype Ontology*, ([25]).

A cellular component may be for instance an anatomical structure, e.g. "dendrite" or "synaptic vesicle" but also a cell or a protein. Note that a "molecule" is defined in the Neuronal Ontology as a set of atoms covalently linked. A molecule cannot contains other molecules. Hence, a protein made up of several subunits, or a polypeptide and a co-enzyme are not "molecules", but "molecular complexes". Although our ontology is built for DopaNet purposes, it can be viewed as a more general "Neuronal Ontology". Therefore, we incorporate terms related to components present (or events taking place) in any neuron, not necessarily DopaNet target cells. In particular, such additions are advised if they clarify some hierarchical relationship.

As described above, a "molecular complex" in DopaNet Neuronal Ontology contains one or several components, also present in the "molecule" branch. It could be considered redundant that all monomeric proteins are represented by two terms, as a "molecule" part of a "molecular complex". However, the meanings of the two branches are different. The "molecule" can be seen as an ideal entity, while the latter would rather represent an actual physical object of the cell. Moreover, the hierarchical structures of the two branches are different. In addition, a lot of proteins have only recently been discovered as functional complexes (e.g. the polymeric G-protein coupled receptors), and more are to be discovered. Finally, the systematic dissociation between the functional molecular complex and its components is handy when it comes to write the Molecular Pages.

### Molecular Pages

The Alliance for Cellular Signaling was a pioneer in designing Molecule Page. Contrary to DopaNet Molecular Pages, their focus is truly a "molecule" rather than a "molecular complex". For instance, an heteropolymeric receptor will not be represented by a Molecule Page, but rather by a collection of Pages, one per subunit.

DopaNet Molecular Pages are highly structured. While this could appear as an obvious choice, it actually comes with a double burden. First, the edition interface has to be sufficiently complex to reflect the underlying structure. This complexity certainly acts as a repellent for the biologist who wish to build a Molecular Page. Second, the high quality required, in particular concerning the annotations, leads to the rejection of a significant portion of the published knowledge. However, we think that a piece of data that cannot be properly annotated is of limited use for the community. For instance, a large amount of pharmacological properties is published without the species. Since those properties vary from one species to the other, one cannot easily re-use the value provided. Similarly, a numerical piece of knowledge cannot be used without caution if one does not know the method used to collect it, and the variability attached to it. Currently, the access to the data is only possible through the web interface. Moreover, although the user is able to search the content of the Molecular Pages using various criteria, the result is always presented as one or several Molecular Pages. However, the DopaNet Web Service should be enriched on a steady pace, and specific pieces of data should be served soon. One can envision interfaces providing precise and meaningful responses to queries like "All Kd for the ligand X of all molecules that bind it", under the form of a list of *K*_*d *_In addition, pieces of quantitative knowledge, like binding or enzymatic reactions, should be provided in standardized format such as the Systems Biology Markup Language [26].

The Molecular Pages are maintained in a distributed fashion, with one or several experts in charge of each complex. Such an approach is mandatory for two reasons. Firstly, the knowledge accumulated by the project will soon become much too large to be handled by one individual, or even one team. Secondly, the level of detail and accuracy sought by the resource is such that only experts can fruitfully mine the adequate literature for relevant information. To extract the simple affinity of a receptor for a ligand can be a daunting prospect. Not only that affinity can be expressed by various parameters with different meaning, *K*_*a*_, *K*_*d*_, *K*_*i*_, *K*_*p*_, *IC*_50_, but all those quantities can only be analyzed in regards of the knowledge about the various states of the complex, and its conformational transitions. The distributed annotation can cause concerns related to peer-validation and quality control. With the help of Nature Publishing Group, a peer review process has been set-up by the Alliance for Cellular Signaling to survey the edition of its Molecule Pages. Such an infrastructure is currently out of reach of DopaNet. However, we ensure that the maintainers are always recognized experts in the fields, or, for members of the EBI group, work in close relation. In addition, we included as much as possible guidance through the constraints imposed by the Page editing environment. That way, any Molecular Page complies with at least a minimal set of quality rules. Such an approach has already been successful in other areas. One of the most striking example is the Debian operating system project ([27]), that maintains around 9000 software packages for 11 computer architectures, with the help of about 1000 developers. The project has been running since 1993, and it is recognized as one of the most robust operating systems.

On the contrary of the Molecular Pages, the Neuronal Ontology is currently developed only by the EBI team. Everyone can contribute by sending their suggestions, but for the sake of coherence the final building is centralized.

## Conclusions

DopaNet Molecular Pages allow to store annotated numerical data about molecular complexes involved in neuronal signaling. Although the Pages are one of the core resources of the DopaNet project, and therefore their focus on the mesotelencephalic dopamine system, the repository should be of widespread utility in the field of Systems Neurobiology. This is also the case of The DopaNet Neuronal Ontology. The resource is in its early stage of development and will benefit much from the feedback of users.

## Availability and requirements

All data contained in the DopaNet Molecular Pages may be copied and redistributed freely, under terms derived from the MIT license [28].

More information about the DopaNet project can be found at the URL .

DopaNet ontology is available at the URL 

DopaNet Molecular Pages are available at the URL



## Authors' contributions

NLN designed the database DTDs and schemas, wrote the XSL and acted as the final editorial authority on Molecule Pages. MD implemented all the edition and validation software, as well as the user interface, including the servers.

## Figures and Tables

**Table 1:** Use of internal and external state references This example (in pseudocode for sake of compactness) shows the how the notions of receptor and ligand are relative to the current Molecular Page. It also makes use of internal and external links to the various states of the interacting complexes. Note that each interaction is coded twice, although the stateMolecule and stateLigand attributes are reversed. Internal links are in italic, while external links are underlined.

molecularPage name="mol1" listOfStates state identifier="greedy" state identifier="neutral" ligand name="mol2" stateLigand="active" property name="Kd" stateMolecule="*greedy*" value mean="10" unit="nanomole per litre" property name="Kd" stateMolecule="*neutral*" value mean="1" unit="micromole per litre"	molecularPage name="mol2" listOfStates state identifier="active" state identifier="inactive" ligand name="mol1" stateLigand="greedy" property name="Kd" stateMolecule="*active*" value mean="10" unit="nanomole per litre" ligand name="mol1" stateLigand="neutral" property name="Kd" stateMolecule="*active*" value mean="1" unit="micromole per litre"
